# Distinct developmental growth patterns account for the disproportionate expansion of the rostral and caudal isocortex in evolution

**DOI:** 10.3389/fnhum.2014.00190

**Published:** 2014-04-08

**Authors:** Christine J. Charvet

**Affiliations:** Department of Psychology, Cornell UniversityIthaca, NY, USA

**Keywords:** cortex, gradient, neurogenesis, development, evolution

## Abstract

In adulthood, the isocortex of several species is characterized by a gradient in neurons per unit of cortical surface area with fewer neurons per unit of cortical surface area in the rostral pole relative to the caudal pole. A gradient in neurogenesis timing predicts differences in neurons across the isocortex: neurons per unit of cortical surface area are fewer rostrally, where neurogenesis duration is short, and higher caudally where neurogenesis duration is longer. How species differences in neurogenesis duration impact cortical progenitor cells across its axis is not known. I estimated progenitor cells per unit of ventricular area across the rostro-caudal axis of the isocortex in cats (*Felis catus*) and in dogs (*Canis familiaris*) mostly before layers VI-II neurons are generated. I also estimated the ventricular length across the rostro-caudal axis at various stages of development in both species. These two species were chosen because neurogenesis duration in dogs is extended compared with cats. Caudally, cortical progenitors expand more tangentially and in numbers in dogs compared with cats. Rostrally, the cortical proliferative zone expands more tangentially in dogs compared with cats. However, the tangential expansion in the rostral cortical proliferative zone occurs without a concomitant increase in progenitor cell numbers. The tangential expansion of the ventricular surface in the rostral cortex is mediated by a reduction in cell density. These different developmental growth patterns account for the disproportionate expansion of the rostral (i.e., frontal cortex) and caudal cortex (e.g., primary visual cortex) when neurogenesis duration lengthens in evolution.

## Introduction

Across species, the frontal and visual cortices expand with positive allometry with respect to many other cortical areas (Bush and Allman, 2004; Kaskan et al., 2005). The disproportionate expansion of the frontal and caudal poles relative to other cortical regions must be important but how do they arise developmentally? Evolutionary changes in the duration of neurogenesis gives rise to species differences in brain size and neuron numbers in adulthood (Finlay and Darlington, 1995; Vaccarino et al., 1999; Striedter, 2005; Finlay, 2008; Striedter and Charvet, 2008; Dyer et al., 2009; Charvet et al., 2012; McGowan et al., 2012; Finlay and Workman, 2013; Workman et al., 2013). However, very little is known about how variation in the duration of neurogenesis across the presumptive isocortex influences the kinetics of progenitor cells and allometry of the rostral and caudal isocortex.

Early in isocortical development, cells proliferate and are located exclusively within the ventricular zone (Boulder Committee, 1970; Bystron et al., 2008). As development progresses, cells continue to undergo cell divisions within the ventricular zone but some cells exit the cell cycle and migrate out of the ventricular zone to become neurons or glia (Rakic, 1974; Tan and Breen, 1993; Tan et al., 1995). Other cells continue to proliferate but migrate out of the ventricular zone to form the subventricular zone, and eventually give rise to neurons or glia (Boulder Committee, 1970; Noctor et al., 2004; Bystron et al., 2008; Molnár, 2011; Kennedy and Dehay, 2012; Molnár and Clowry, 2012). At some point, many of the cells switch from undergoing symmetrical proliferative to asymmetrical cell divisions, where one daughter cell is non-proliferative and the other cell is proliferative (Dehay and Kennedy, 2007). As more and more cells exit the cell cycle, the cell cycle rate lengthens (Takahashi et al., 1995; Charvet and Striedter, 2008) until eventually the progenitor population wanes and most of neurogenesis ends (Caviness et al., 2003). Extending neurogenesis involves altering a coordinated sequence of developmental changes in cell cycle kinetics (Gohlke et al., 2007; Charvet et al., 2012).

A prolongation in the duration of neurogenesis entails extending the length of time in which cells proliferate and a delay in the switch from proliferative symmetrical to asymmetrical cell divisions (Dehay and Kennedy, 2007; Charvet and Striedter, 2011), thereby expanding the proliferative pool. Delaying cell cycle exit also entails delaying the decline in the cell cycle rate (Takahashi et al., 1995; Charvet and Striedter, 2008), which implies cells cycle faster for longer. Delays in cell cycle exit and delays in the decline in the cell cycle rate should exponentially increase the number of proliferative cells in the ventricular and subventricular zone and cause an amplification of neurons in adulthood.

**Figure 1:**
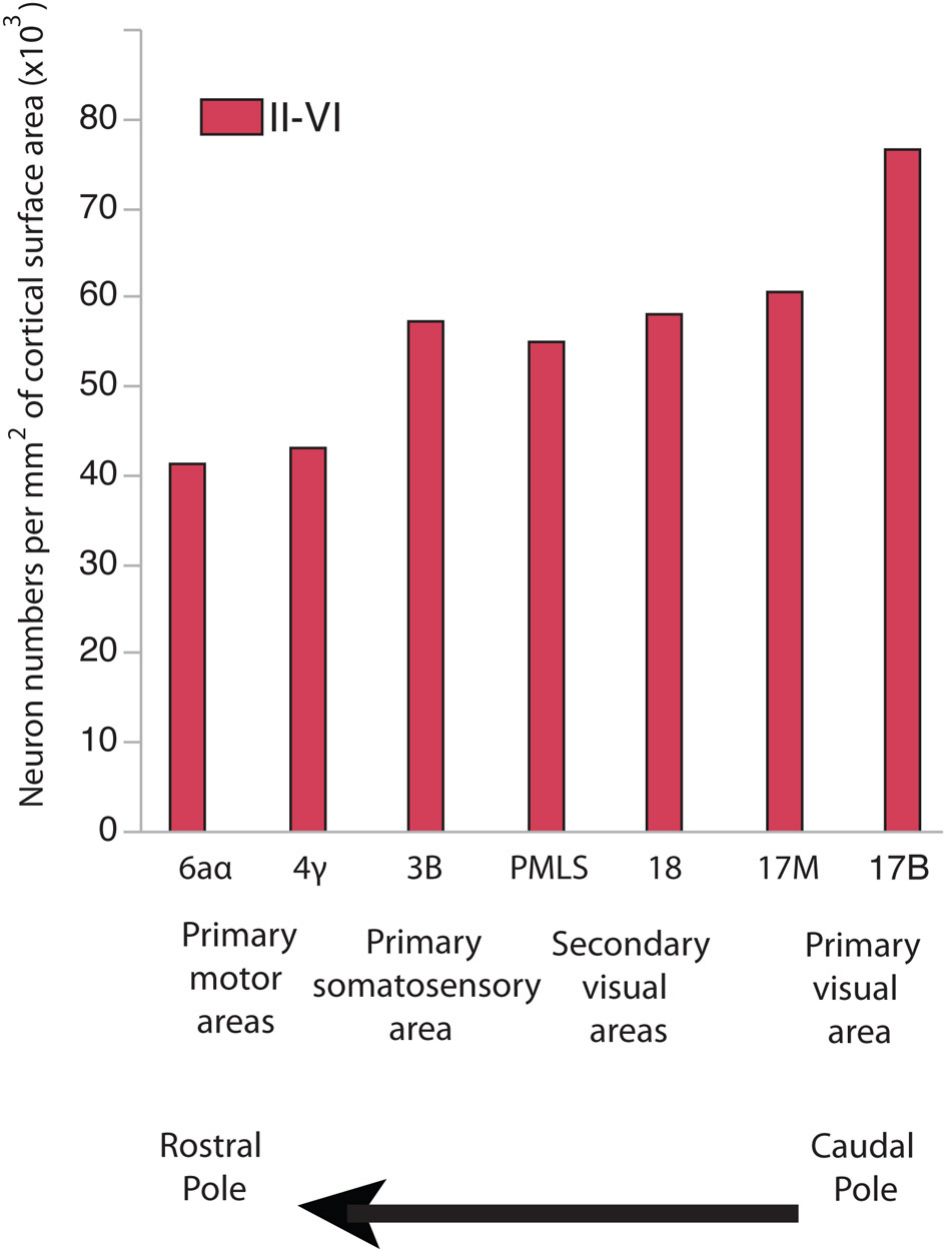
**Neuron numbers per unit of cortical surface area in different cortical areas in the cat**. Neurons per unit of cortical surface area are high toward the caudal pole and systematically decrease toward the rostral pole. 17B and 17M stand for the binocular and monocular portion of area 17, respectively. PMLS: posteromedial suprasylvian area. These data are from Beaulieu and Colonnier (1989).

In development, there are gradients in neurogenesis duration across the isocortex (Rakic, 1974, 2002; Luskin and Shatz, 1985; Smart, 1985; Bayer and Altman, 1990, 1991; Smart et al., 2002). Neurogenesis onset occurs simultaneously throughout the isocortex. Terminal neurogenesis varies substantially between its rostral and caudal pole in some species (Rakic, 1974, 2002; Workman et al., 2013). That is, precursor cells that give rise to layer VI-II neurons undergo their final rounds of cell divisions earlier in the rostral pole than in the caudal pole (Rakic, 1974, 2002). The extension of neurogenesis duration in the caudal pole entails that cells undergo more rounds of cell divisions, delay the switch from symmetric proliferative to asymmetric cell divisions and delay the decline in the cell cycle rate (Charvet and Striedter, 2008), which should cause an exponential amplification of neurons in the caudal pole relative to the rostral pole. In line with this prediction, there are more neurons per unit of cortical surface area in the caudal pole than in the rostral pole of primates, cats and some rodents (Figure 1; Beaulieu and Colonnier, 1989; Shankle et al., 1998; Collins et al., 2010; Cahalane et al., 2012; Charvet et al., 2013; Ribeiro et al., 2013).

According to the radial unit hypothesis, the tangential locations of pyramidal neurons within the adult isocortex are determined by the relative positions of their precursors within the isocortical proliferative zone (Luskin et al., 1988; O'Rourke et al., 1992; Tan and Breen, 1993; Rakic, 1995; Tan et al., 1995; Ma et al., 2013). After exiting the cell cycle, neurons migrate radially and their location across cortical layers is thought be determined by their date of birth (Rakic, 1995). Rakic suggested that the tangential expansion of proliferating cells is a mechanism to increase the number of radial columnar units in evolution (Rakic, 1995, 2000). Altering cell cycle kinetics by extending neurogenesis duration would tangentially expand the cortical proliferative zone and increase the number of radial columnar units superficial to the progenitor cells that generated them, causing the isocortex to expand primarily tangentially rather than in height (Rakic, 1995; Hutsler et al., 2005).

As neurogenetic schedules lengthen and overall brains expand, neurogenesis occurs earlier in the frontal cortex than in other regions. The frontal cortex also expands disproportionately relative to many other cortical regions (Bush and Allman, 2004) and most of this expansion is tangential (Hutsler et al., 2005). The positive allometry of the frontal cortex may seem surprising. It would be expected that a shortened duration of neurogenesis should generate fewer cell divisions and generate fewer progenitor cells, which should cause less tangential expansion of precursor cells within the proliferative zone and fewer radial columnar units by adulthood. The developmental mechanisms that give rise to the positive allometry of the frontal cortex relative to other cortical areas is therefore not clear.

Investigating the effects of neurogenesis duration on progenitor cell numbers may help elucidate how the rostral and caudal pole expand with positive allometry with respect to other cortical areas in evolution. The aim of this study is to examine the consequences of neurogenesis extension on progenitor cell numbers across the rostro-caudal axis of the developing isocortex. Cats and dogs were selected because cats exhibit a gradient in neurons per unit of cortical surface area across the rostro-caudal axis in adulthood (Figure 1) and because the duration of isocortical neurogenesis in dogs is longer than in cats (Table 1). I quantified proliferating cells in the ventricular zone and subventricular zone (if present) across the rostro-caudal axis of the isocortex in these two species shortly before and during isocortical neurogenesis (Luskin and Shatz, 1985; Workman et al., 2013). The main finding from the present study is that progenitor cells in the caudal pole expand more tangentially with a concomitant increase in progenitor cells in dogs compared with cats. However, progenitor cells in the rostral pole expand more tangentially but without a concomitant increase in cell numbers in dogs compared with cats. This is mostly mediated by a reduction in cell density within the rostral proliferative zone. These distinct growth patterns account for differences in the cellular constituents underlying the allometries of the rostral and caudal pole of the isocortex relative to other cortical areas in evolution.

**Table 1:** **The timing of neural events in cats and dogs is expressed in embryonic days (ED) after conception**.

**Neural events**	**Cat (ED)**	**Dog (ED)**	**Dog NP**	**Dog P**	**Cat NP**	**Cat P**
Preplate emerges in the isocortex	18.75	21.75	8.5 mm (30)	9 mm (39, 40)	8 mm (45, 48, 122)	9 mm (B)
Post-proliferative zone emerges in the cerebellum	18.75	22.5	9 mm (39, 40)	10 mm (12)	8 mm (45, 48, 122)	9 mm (B)
Post-proliferative zone emerges in the superior colliculus	20.5	24.5	10 mm (12)	13.5 mm (95, 97)	9 mm (B)	12 mm (53)
Subventricular zone emerges in the ganglionic eminence	21.5	27	13.5 mm (95, 97)	17 mm (74)	10 mm (54)	16 mm (19, 109)
Cortical plate emerges in the rostral pole of the isocortex	22.75	28	15 mm (99)	19 mm (131)	13 mm (79)	18 mm (125)
Cortical plate emerges in the caudal pole of the isocortex	25.75	30.25	19 mm (120)	25 mm (255)	16 mm (19, 109)	26 mm (66)
Subventricular zone emerges in the rostral pole of the isocortex	26.25	30.75	21 mm (121)	25 mm (255)	18 mm (125)	26 mm (66)
Subventricular zone emerges in the caudal pole of the isocortex	29.25	32.75	25 mm (255)	32 mm (108)	26 mm (66)	33 mm (82)
Mitral cell layer emerges in the olfactory bulb	31.75	38.75	40 mm (106A)	67 mm (126)	33 mm (82)	41 mm(60)

Crown rump length is expressed in millimeters (mm) and identification number of embryos used to estimate developmental events are in parantheses. The timing of an event was estimated by examining when an event was not present (NP) and when the transformation in brain anatomy was present (P).

## Materials and methods

Nissl-stained sections of embryonic cats (*Felis catus*) and beagles (*Canis familiaris*) at various stages of development were borrowed from the Cornell School of Veterinary Medicine Embryological Collection (Table 1). This embryological collection is an ideal resource to investigate how progenitor cells vary across the cortical proliferative zone and across species because it houses a large number of embryological specimens and these specimens are sectioned thin enough so that progenitor cells within the presumptive isocortex may be quantified.

Crown rump length information was converted to age in days according to a previously published study that had utilized the Cornell School of Veterinary Medicine Embryological Collection (Evans and Sack, 1973). That is, I regressed crown-rump length vs. embryonic days (ED) for each species (Evans and Sack, 1973) in order to estimate age for each specimen in the collection. In order to compare progenitor cells at equivalent stages of development in these two species, I estimated and compared the timing of neural events in dogs and cats at various stages of development. Neural events are defined as rapid transformations in brain morphology and these neural events were chosen to cover the time course in which the progenitor pool populations were estimated (Table 2; Workman et al., 2013). Coronal, sagittal and horizontal sections of specimens were used in these analyses.

**Table 2:** **The ID of the specimens, crown-rump length (CRL) information and estimated ages in embryonic days (ED) are listed**.

**Species**	**Specimen ID**	**CRL (mm)**	**Age (ED)**	**Tissue thickness**
Cat	101	7	18	10 μm
Cat	48	8	18.5	10 μm
Cat	45	8	18.5	10 μm
Cat	122	8	18.5	10 μm
Cat	47	9	20	10 μm
Cat	53	12	22	10 μm
Cat	56	16	23.5	15 μm
Cat	124	17.5	24.5	10 μm
Cat	125	18	25	10 μm
Cat	66	26	29	15 μm
Cat	87	60	39.5	15 μm
Dog	112	7	19.5	10 μm
Dog	40	9	22	10 μm
Dog	156	10.5	24	10 μm
Dog	117	12	25	10 μm
Dog	71	12	25	10 μm
Dog	77	13	26	10 μm
Dog	97	13.5	26	10 μm
Dog	28	17	28	10 μm
Dog	120	19	29	15 μm
Dog	108	32	34	15 μm
Dog	126	67	42	20 μm

**Figure 2:**
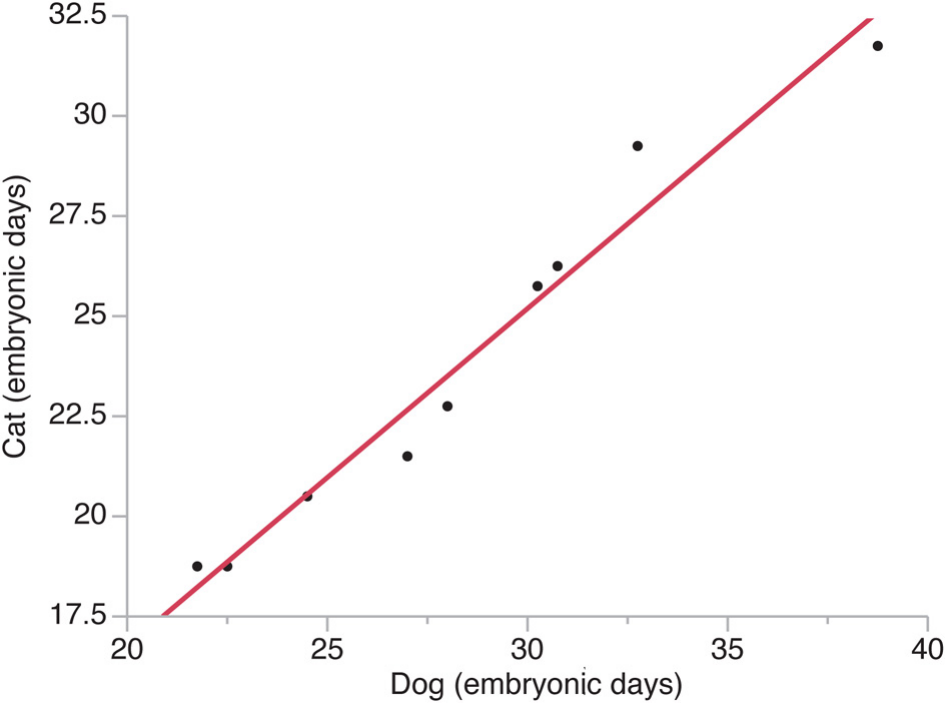
**The timing of neural events in cats is regressed against the timing of neural events in dogs**. Time is expressed in embryonic days after conception with the day of conception designated as embryonic day 0. Neural events are listed in Table 1.

The timing of a neural event was estimated by examining when a transformation in brain morphology was not present and when the transformation in brain morphology had occurred. The timing of a neural event was determined by fitting a spline regression through the crown rump length versus age in each species and finding the corresponding age mid-way between when the event is not present and when it is present. Neural event timing in cats was regressed against neural event timing in dogs (Figure 2) and the dog's developmental schedule was converted to the cat's developmental schedule. Gestational length is slightly shorter in cats than in dogs with birth occurring 65 days after conception in cats and 60 days in dogs (Evans and Sack, 1973; Workman et al., 2013). However, the timing of neural milestones in dogs is longer than in cats (Table 1). That is, cats are born somewhat more mature than dogs, and mature more quickly, although these are small differences in developmental timing considering the range of developmental duration within mammals (Workman et al., 2013).

**Figure 3:**
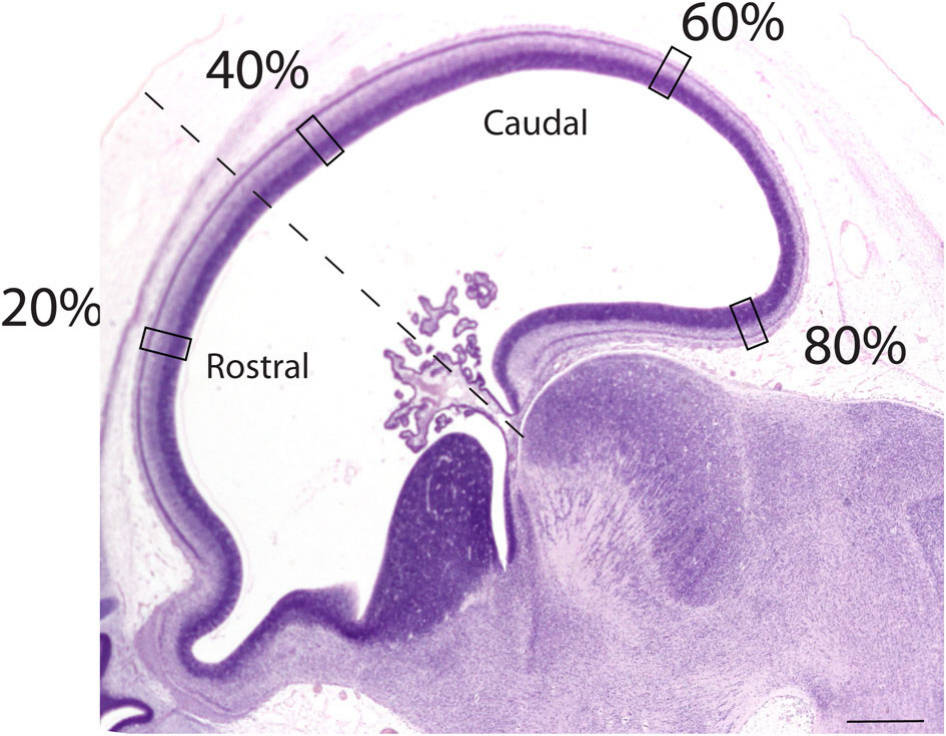
**Representation of a sagittal section used to estimate the progenitor cells along the rostro-caudal axis of the cerebral cortex**. The ventricular length was estimated and divided into percentiles. Estimates of the progenitor cell numbers at every 20, 40, 60, 80% along the ventricular surface were made. Represented here is a sagittal section of an embryonic day 34 dog (CRL 32 mm). The dashed lines mark the boundary between the rostral and caudal cortex. Scale bar is 1 mm.

Sagittal sections of embryonic specimens were used to estimate progenitor cells per unit of ventricular area and these specimens were selected to cover time points before cortical layer neurons are generated. The developing cerebral cortex exhibits curvature across the medio-lateral and rostro-caudal axes. Estimating progenitor cell numbers per ventricular area may be compromised if the area under investigation exhibits high curvature. Therefore, a select area that exhibits minimal curvature along the medial to lateral axis was selected. Areas rostral and dorsal to the olfactory bulb were selected in both species (Figure 3). Adjacent sections and coronal sections of other embryos in the collection were examined to ensure that the selected regions are not compromised by high curvature. The ventricular length was estimated across the rostro-caudal axis of the developing cerebral cortex to determine the location of the sites of interest. The ventricular length was subdivided into percentiles to apply counting frames. Four equidistant measurements were made along the rostro-caudal axis (Figure 3) and two sections were sampled per specimen. The selected regions in this study coincide with areas where the variation in neurons under a unit of cortical surface area is maximal in adulthood (Beaulieu and Colonnier, 1989; Collins et al., 2010; Cahalane et al., 2012; Charvet et al., 2013).

The Abercrombie correction factor was used to estimate cortical progenitor cells per ventricular area. Cell estimates were corrected following Abercrombie's correction factor: P = A(M)/(L+M) where P is the corrected number of cells estimates, A is the crude cell count, M is the thickness of the section and L is the diameter of the cell. In development, the proliferative pool (i.e., ventricular zone, subventricular zone) is densely packed with cells, which makes it difficult to count individual cells within these proliferative zones. However, ventricular and subventricular zone cells can be counted if the sections are thin enough (e.g., 10 μm thick). In these thin sections it is virtually impossible to employ the z-plane guard zones that is necessary for the optical dissector method (Williams and Rakic, 1988). However, the cell bodies of these proliferative cells are spherical so that the Abercrombie's correction factor may be applied to correct for over-counting (Abercrombie, 1946).

Sections were examined with a Leitz Diaplan microscope and a Neurolucida imaging system with a mechanical stage (Mircrobrightfield, Inc., Colchester, VT, USA). Counting frames were aligned with the ventricular surface and were 41 microns in width although the height varied according to the thickness of the ventricular zone and subventricular zone, if present. Very few of these estimates include the subventricular zone because most of the specimens were selected at ages where the subventricular zone had not yet emerged. Abrupt changes in cell density in the proliferating pool (i.e., ventricular and subventricular zone) were used as the criterion to define the proliferative region. Two sides of the counting frames served as exclusion lines to avoid over-estimating cells. In every sample, the area of 4–10 somas was measured and diameters of the cells were estimated. I applied a line radial to the ventricular surface and measured the soma of those cells or a subset of cells that intersected the line. Two samples per region were selected for each embryo. Those values were averaged to represent one value per cortical location per embryo. Progenitor cells per unit of ventricular surface area are an estimate of the total number of proliferating cells under a unit of ventricular surface area. Progenitor cells per unit of ventricular surface area vary according to the density and thickness of the progenitor pool. I compared the progenitor cells per unit of ventricular surface area, cell densities, and thickness of the proliferative pool in dogs and cats. I regressed the variable of interest for each species against their equivalent developmental timetable. Statistical analyses were performed in JMP11.

Estimating the surface length across the rostro-caudal axis of the progenitor pool is challenging considering the lack of anatomical boundaries within the growing presumptive isocortex. However, anatomical landmarks within other regions may be used to examine how the cortical progenitor cells grow differentially across the rostro-caudal axis. Here, the choroid plexus was used as an anatomical landmark to distinguish the rostral and caudal cortex. The rostral boundary of the isocortex was distinguished from the olfactory bulb by the presence of the cortical plate. The caudal region here includes the medial pallium (e.g., hippocampus). The boundary between the rostral and caudal region of the cortex was done by drawing a line between the olfactory bulb and the choroid plexus and then drawing a line perpendicular to that line. The region where the line intersected the presumptive cortex was considered the boundary between the rostral and caudal cortex. The rostro-caudal length of the ventricular surface was estimated in the frontal and caudal regions. A subset of specimens was used in this analysis because only specimens in which these anatomical landmarks were clearly visible were selected.

The Abercrombie method is limited in that estimates may be biased if cell soma size differs between groups (Hedreen, 1988; von Bartheld, 2002). To address this potential issue, I averaged the cell diameter estimates for each embryo and compared the average cell diameter between cats and dogs. A two-sample t test, assuming unequal variance, shows that the average cell diameter (*x* = 1.73, *SD* = 0.12; *n* = 11) of dogs is not significantly different from that of cats (*x* = 1.75, *SD* = 0.09; *n* = 11; *t* = 0.58, *p* > 0.05). The differences in progenitor cell numbers observed between these two species are unlikely to be biased by differences in cell size.

## Results

The timing of neural events was estimated within the presumptive isocortex and within other brain regions in cats and dogs (Figure 2). In these species, developmental milestones such as the emergence of the cortical plate and the isocortical subventricular zone emerge earlier in the rostral pole than in the caudal pole in cats and in dogs (Table 1). That is, developmental milestones occur over a shorter time interval in the rostral pole than in the caudal pole. These findings are consistent with the notion of a gradient in developmental duration where neuronal maturation occurs earliest rostrally during isocortical neurogenesis.

A natural-logged regression was fit to neural event timing in both species (*y* = 1.006 × −0.196; adj *R*^2^ = 0.96). This regression was used to translate the timing of brain maturation in order to compare progenitor cell estimates between cats and dogs (Figure 2). In cats, neurogenesis of cortical layers starts on ED 28 and ends on ED 57 in the developing primary visual cortex (Luskin and Shatz, 1985; Workman et al., 2013), which would equate to ED 33 to ED 68 in dogs. Therefore, the duration of cortical layer neurogenesis in the beagle is extended by 6 days relative to that of cats. The observation that the beagle's brain (brain weight = 84.3 g; body weight = 14.8 kg) is nearly three times bigger than that of the cat (brain weight = 29.6 g; body weight = 3.7 kg; Bronson, 1979) is consistent with the notion that a lengthened duration of neurogenesis generates more isocortical neurons in dogs than in cats.

**Figure 4:**
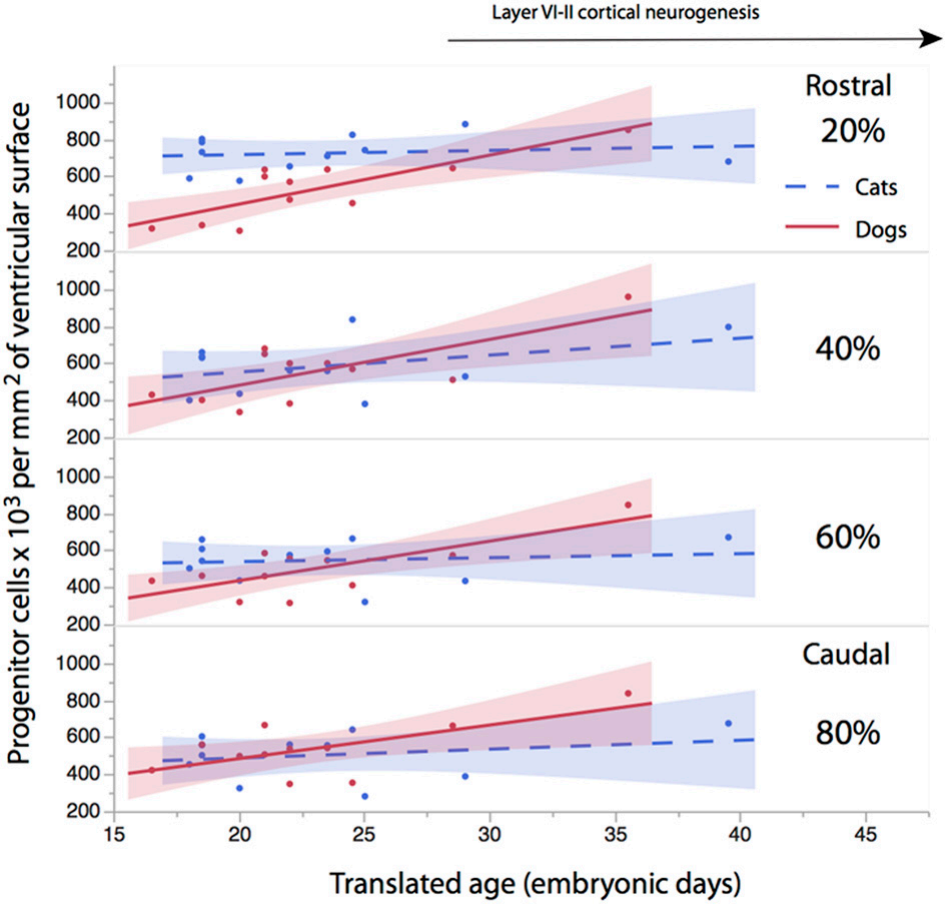
**The progenitor cells per mm^2^ of ventricular surface are regressed against translated age at different sites across the rostro-caudal axis (i.e., 20, 40, 60, 80%)**. Twenty percentage stands for the most rostrally-selected region and 80% stands for the most caudally selected region. A linear regression and 95% confidence intervals are fit for each of the selected sites for each species. The duration of cortical layers VI-II neurogenesis in the primary visual cortex are shown for the cat.

The progenitor cells per mm^2^ of ventricular surface were estimated at equidistant sites across the rostro-caudal axis (Figure 3). The age of cats ranges between ED18 and ED 39.5 and the age of dogs ranges between ED 19.5 and ED 42. Most of these ages cover time points before cortical layer neurons are generated. At most ages examined, the subventricular zone has not yet emerged (Table 1). Progenitor cells per mm^2^ of ventricular surface increase at a faster rate in dogs than in cats (Figure 3, Table 3). The progenitor cells per mm^2^ of ventricular surface overlap extensively in all selected cortical sites with the exception of the most rostrally selected region (Figure 4; designated as 20%). Toward the rostral pole, dogs exhibit fewer progenitor cells per unit of ventricular area compared with cats (Figure 4).

**Table 3:** **Summary of statistical parameters for progenitor cells per ventricular surface mm^2^ regressed against translated age in cats and dogs**.

**Rostro-caudal location**	**Linear regression**	**Intercept *SE***	**Slope *SE***	**Adjusted *R*^2^**	**Intercept *t* ratio; *p*-values**	**Slope *t* ratio; *p*-values**
20%-Cat	*Y* = 2.27x + 675.59	119.39	4.94	−0.08	5.66; *p* < 0.05	0.46; *p* = 0.66
40%-Cat	*Y* = 9.12x + 374.79	171.64	7.11	0.06	2.18; *p* = 0.57	1.28 *p* = 0.23
60%-Cat	*Y* = 2.15x + 499.75	139.85	5.79	−0.09	3.57; *p* < 0.05	0.37; *p* = 0.73
80%-Cat	*Y* = 4.78x + 396.74	156.65	6.49	−0.05	2.53; *p* < 0.05	0.74; *p* = 0.48
20%-Dog	*Y* = 26.46x − 74.71	148.12	6.29	0.62	−0.50; *p* = 0.63	4.2; *p* < 0.05
40%-Dog	*Y* = 24.74x − 8.49	181.09	7.69	0.48	−0.05; *p* = 0.96	3.21; *p* < 0.05
60%-Dog	*Y* = 14.71x + 21.31	146.74	6.23	0.52	0.10; *p* = 0.92	3.42; *p* < 0.05
80%-Dog	*Y* = 18.15x + 126.19	163.96	6.97	0.36	0.77; *p* = 0.46	2.61; *p* < 0.05

To determine whether there are significant differences in the progenitor cells per unit of ventricular area between cats and dogs, I compared the 95% confidence intervals of per mm^2^ of ventricular surface regressed against translated age in both species. In the rostral isocortex, the 95% confidence intervals of the progenitor cells in dogs (*y* = 26.46 × −74.71; slope *SE* = 6.29; intercept *SE* = 148.12; adj *R*^2^ = 0.62) fall below the lower 95% confidence interval of those for cats between ED 17 and ED 23 (Figure 4, *y* = 2.27x + 675.59; slope *SE* = 4.94; intercept *SE*: 119.39; adj *R*^2^ = −0.08; Table 3). This age range in cats equates to ED19 and ED 28 in dogs. These findings demonstrate that the progenitor cells per unit of ventricular area in the rostral region of dogs is smaller than that of cats by the time layer VI-II neurons are generated.

**Figure 5:**
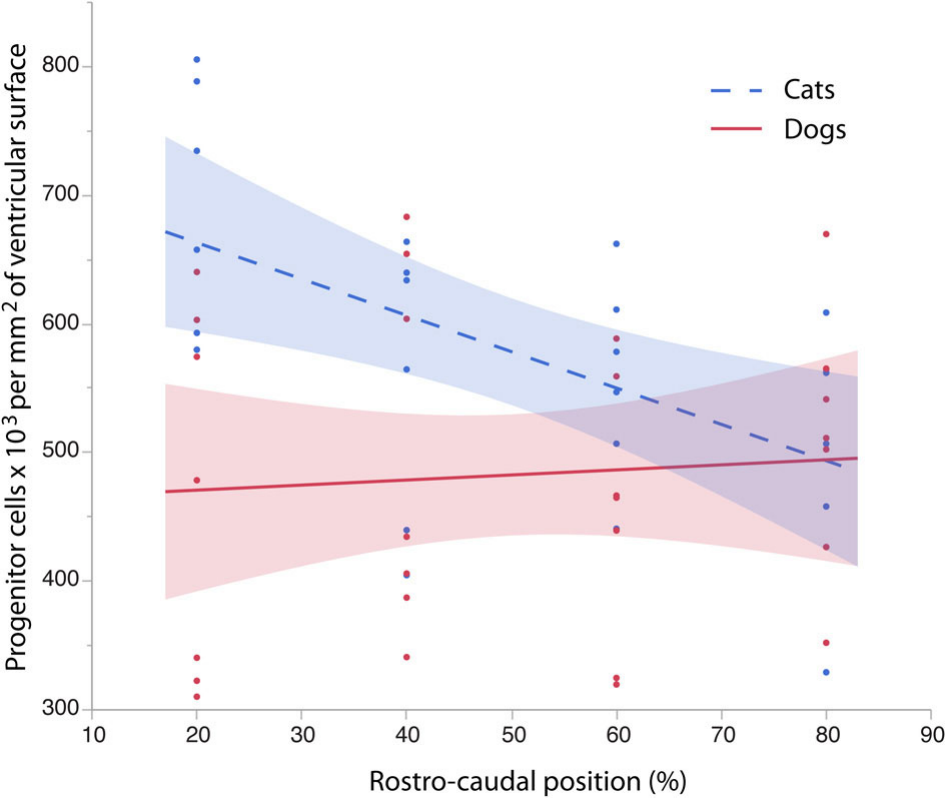
**The progenitor cells per mm^2^ of ventricular surface are regressed against the rostro-caudal axis of the cortex in cats and dogs at equivalent stages of development**. Progenitor cells for each selected sites were selected between ED 16 to ED 22 in cats (=6) and between ED 19.5 to ED 26 in dogs (*n* = 6) to cover time points before the cortical plate emerges. Progenitor cells per ventricular area gradually decrease between the rostral and caudal regions in the cat whereas progenitor cells per ventricular area vary little over the rostral to caudal axis in the dog. These findings demonstrate that there are species differences in progenitor cells per ventricular area by the time layer VI-II neurons are born.

To further investigate the differences in progenitor cells across the rostro-caudal axis of the presumptive isocortex, I regressed the progenitor cells per unit of ventricular area against the rostro-caudal axis in cats (ranging in age between ED 18 and ED 22; *n* = 6) and in dogs (ranging in age between ED 19.5 and ED 26; *n* = 6; Figure 5). At these ages, a cortical plate and a subventricular zone have not yet emerged (Table 1). Such an analysis shows that the progenitor cells per mm^2^ of ventricular surface significantly decrease from the rostral to caudal pole in cats (*y* = −2.83x + 719.64; adj *R*^2^ = 0.28; *n* = 24; slope t ratio = −3.16; *p* < 0.05; intercept t ratio = 14.7; *p* < 0.05). In contrast, the progenitor cells per mm^2^ of ventricular surface area do not vary significantly across the rostral to caudal axis in dogs (*y* = 0.39x + 462.94; adj *R*^2^ = −0.03; *n* = 28; slope t ratio = 0.38; *p* = 0.70; intercept *t* ratio = 8.26; *p* < 0.01). In the rostral cortex, the 95% confidence intervals derived from these two regressions do not overlap. These findings show that progenitor cells per mm^2^ of ventricular surface is smaller in dogs than in cats in the rostral cortex before layers VI-II neurons are generated.

**Figure 6:**
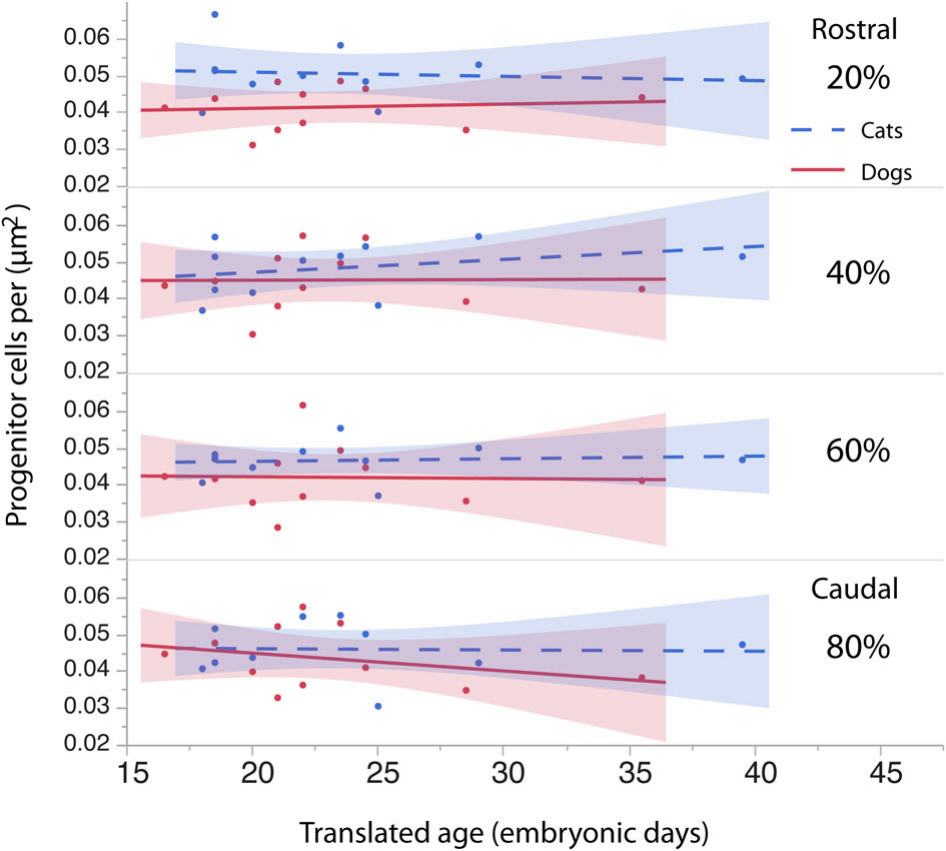
**The density of the progenitor cells is regressed against translated age in cats and dogs**. The cell density in the rostral region of the cortex (i.e., 20%) in dogs is slightly smaller than it is in cats. The cell density in other regions of the cortex (i.e., 40, 60, 80%) is similar in dogs and cats.

**Figure 7:**
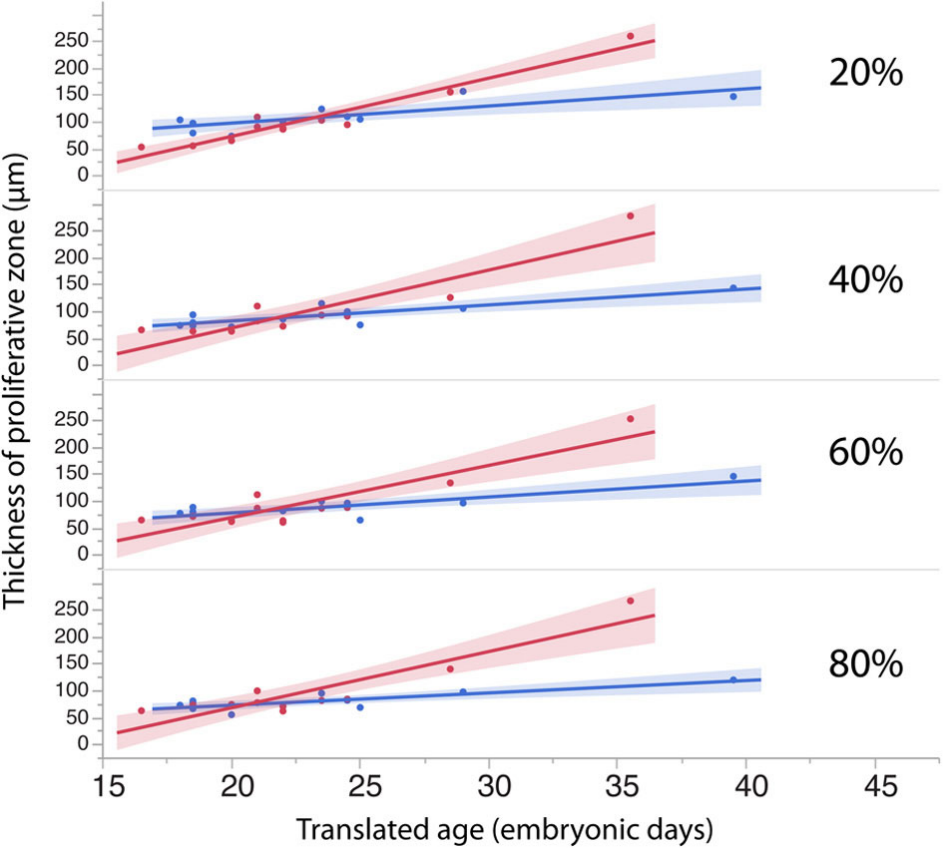
**The thickness of the progenitor population in rostral (20%) and more caudal regions (60–80%) of the cortex have a tendency to increase more over time in dogs than in cats**.

To investigate whether cell densities or thickness of the progenitor cell population account for the differences in progenitor cells per mm^2^ of ventricular surface, cell densities (Figure 6, Table 4) and thickness measurements (Figure 7, Table 5) were regressed against translated age in each species. These regressions show that some of the differences in progenitor cells per unit of ventricular area between cats and dogs may be due to differences in cell density in the rostral cortex. This is evident from the observation that the cell densities in the most rostrally selected region in the presumptive cortex (20%) are smaller in dogs than in cats (Figure 6).

**Table 4:** **Summary of statistical parameters for the cell density of the proliferative zone regressed against translated age in cats and dogs**.

**Rostro-caudal location**	**Linear regression**	**Intercept *SE***	**Slope *SE***	**Adjusted *R*^2^**	**Intercept *t* ratio; *p*-values**	**Slope *t* ratio; *p*-values**
20%-Cat	−1 × 10^−4^ + 0.05	9 × 10^−3^	4 × 10^−4^	−0.10	5.70; *p* < 0.05	−0.30; *p* = 0.77
40%-Cat	3 × 10^−4^x + 0.04	0.01	3 × 10^−4^	−5 × 10^−3^	4.64; *p* < 0.05	0.98; *p* = 0.35
60%-Cat	7 × 10^−5^x + 0.04	6 × 10^−3^	2 × 10^−4^	−0.10	7.54; *p* < 0.05	0.29; *p* = 78
80%-Cat	−3 × 10^−5^ + 0.04	0.01	4 × 10^−4^	−0.11	5.2; *p* < 0.05	−0.09; *p* = 0.93
20%-Dog	1 × 10^−4^x + 0.03	0.01	3 × 10^−4^	−0.10	4.41; *p* < 0.05	0.30; *p* = 0.77
40%-Dog	1 × 10^−5^x + 0.04	0.01	5 × 10^−4^	−0.11	3.7; *p* < 0.05	0.03; *p* = 0.97
60%-Dog	4 × 10^−5^x + 0.04	0.01	5 × 10^−4^	−0.11	3.3; *p* < 0.05	−0.09; *p* = 0.93
80%-Dog	−5 × 10^−4^x + 0.05	0.01	5 × 10^−4^	−0.01	4.66; *p* < 0.05	−0.97; *p* = 0.36

**Table 5:** **Summary of statistical parameters of the thickness of the proliferative zone regressed against translated age in cats and dogs**.

**Rostro-caudal location**	**Linear regression**	**Intercept *SE***	**Slope *SE***	**Adjusted *R*^2^**	**Intercept *t* ratio; *p*-values**	**Slope *t* ratio; p values**
20%-Cat	3.19x + 34.86	19.40	0.80	0.60	1.80; *p* = 0.11	3.97; *p* < 0.05
40%-Cat	2.96x + 24.16	15.04	0.62	0.68	1.61; *p* = 0.14	4.76; *p* < 0.05
60%-Cat	2.97x + 19.57	16.12	0.67	0.65	1.21; *p* = 0.26	4.45; *p* < 0.05
80%-Cat	2.3x + 27.85	12.88	0.53	0.64	2.16; *p* = 0.06	4.31; *p* < 0.05
20%-Dog	10.85x-142.97	23.46	1.00	0.92	−6.09; *p* < 0.05	10.88; *p* < 0.05
40%-Dog	10.80x-145.70	39.14	1.66	0.80	−3.72; *p* < 0.05	6.49; *p* < 0.05
60%-Dog	9.71x-124.03	37.30	1.58	0.78	−3.32; *p* < 0.05	6.13; *p* < 0.05
80%-Dog	10.47x-140.29	37.25	1.58	0.81	−3.77; *p* < 0.05	6.61; *p* < 0.05

**Figure 8:**
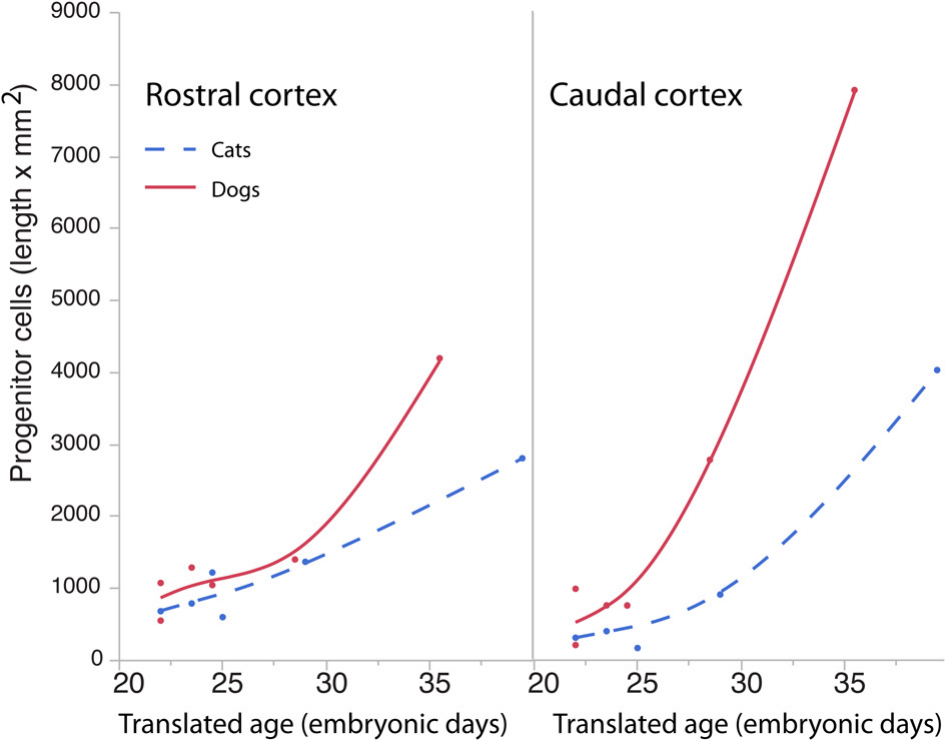
**Progenitor cells in the rostral and caudal regions of the cortex are regressed against translated age in dogs and cats**. Estimated progenitor cells per mm^2^ were multiplied by the length of the ventricular surface in the rostral or caudal region of the cortex. In the rostral cortex, the progenitor cells are roughly similar in both species but increase in the dog compared with the cat at the latest ages examined. In the caudal cortex, the progenitor cells are slightly higher in dogs than in cats at the earliest stages examined and become roughly three times more numerous in the dog compared with the cat at the latest ages examined.

**Figure 9:**
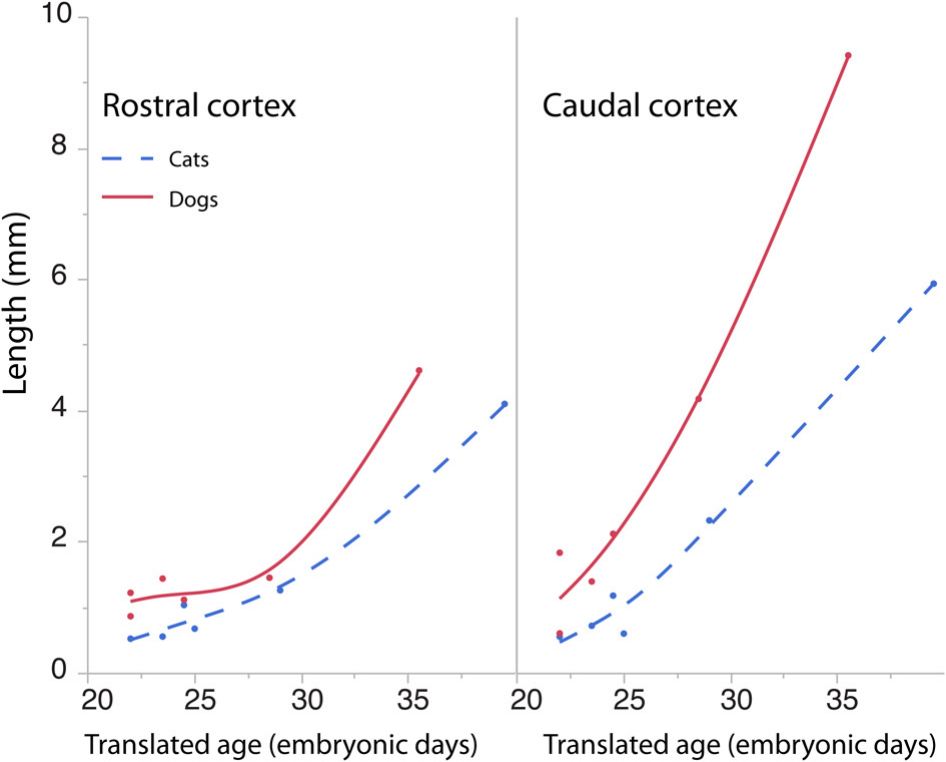
**The cortical ventricular length was divided into a rostral and caudal region in cats and dogs**. The length of the rostral and caudal cortex becomes larger in dogs than in cats toward the latest ages examined. The rostral cortex becomes roughly twice as long in dogs compared with cats and the caudal cortex becomes three times longer in dogs compared with cats at the latest ages examined.

The length of the rostro-caudal ventricular surface and progenitor cell numbers were estimated to determine how cortical progenitor cells grow differentially across the rostro-caudal axis. Progenitor cells were estimated in the rostral and caudal cortices by multiplying the progenitor cells per mm^2^ by the length of the rostro-caudal ventricular surface. Figure 8 shows the resultant estimates of the progenitor cells within the rostral and caudal cortex. Ventricular length estimates may be used to compare the tangential expansion of the ventricular surface area over time in cats and dogs. Figure 9 shows the ventricular length estimates of the rostral and caudal cortex in both species. Such an analysis shows that the rostral and caudal region contain slightly more cells in dogs than in cats at early stages examined. At the latest stages examined, the progenitor cells in the rostral cortex become roughly twice as numerous in dogs compared with cats but progenitor cells in the caudal cortex become roughly three times more numerous in dogs than in cats (Figure 8). That is, the difference in progenitor cells between these two species is greater in the caudal region than in the rostral region by the time layers VI-II neurons are generated. Taken together, the cortical progenitor cells in the caudal region expand more tangentially and in cell numbers in dogs compared with cats. However, the cortical progenitor pool in the rostral region grows more tangentially without a concomitant increase in cell numbers in dogs.

## Discussion

The aim of this study was to determine how lengthened neurogenetic schedules influence cortical progenitor cell numbers across its rostro-caudal axis by the time layers VI-II neurons are generated. The findings from the present study demonstrate that there are different developmental growth patterns accounting for the allometric variation in the size of the rostral and caudal cortex in evolution. The cortical proliferative zone grows tangentially rostrally and caudally but progenitor cells increase more in numbers in the caudal region than in the rostral region in dogs compared with cats.

### Limitations

Although the locations used to estimate the progenitor cells were selected in order to minimize the potential impact of cortical curvature on progenitor cell estimates, it is possible that these estimates may be subject to some distortion due to curvature. Absolute numbers should therefore be interpreted with caution. However, the main aim of this study is to determine the variation in the progenitor cell numbers between species rather than determining absolute values of the progenitor pool populations.

I here do not use the optical dissector method, which is the method of choice when estimating cell numbers (Williams and Rakic, 1988; Williams et al., 2003). The progenitor cells are so densely packed that it would be impossible to employ the optical fractionator method, which requires a guard zone along the z plane (Gardella et al., 2003). The Abercrombie correction factor relies on cell diameter to correct for over-counting. Comparing cell number estimates may be biased if the size of the particles to be counted differs between groups (von Bartheld, 2002). No differences in cell size were observed between the two groups, suggesting that cell size is unlikely to be a source of bias.

One other important caveat to consider in interpreting findings from the present study is that the cortical progenitor cells grow differentially across the axes of the isocortex. The equidistant areas selected in the embryonic cortical progenitor pool population may not necessarily represent the same equidistant sites throughout development and in adulthood. Moreover, the rostro-caudal ventricular length estimates rely on anatomical landmarks. These anatomical landmarks may vary in their relative location in development and may therefore change their position relative to the expanding presumptive isocortex. However, the aim of this analysis was to compare growth in progenitor cells between species rather than to determine absolute growth rates.

Whether the findings from the present study apply widely to mammals is not yet clear (Herculano-Houzel et al., 2011, 2013; Neves et al., 2014). Very little is known about how the timing of cell proliferation gives rise to cortical variation in taxonomic groups other than in rodents and primates. A further investigation of species from a wider range of taxonomic groups would help elucidate how variation in cell cycle kinetics gives rise to diversity in cortical structures across mammals.

### Evolutionary changes in founder cells of the isocortex

Layer VI-II neurogenesis duration in the caudal pole of dogs is estimated to be 6 days longer than in cats. This difference in neurogenesis duration is not nearly as large as it is in other species such as a rhesus monkey and a rat where there is an approximate difference of 56 days in duration in terminal neurogenesis in layer VI-II in the caudal pole between these two species. Nevertheless, extending layer VI-II neurogenesis by 6 days should have a large impact on layer VI-II neuron numbers between cats and dogs in adulthood.

There is a gradient in the duration of neurogenesis across rostro-caudal axis of the presumptive isocortex with neurons born later in the caudal pole than in the rostral pole. Because dogs have a lengthened neurogenetic schedule relative to cats, a longer duration of neurogenesis implies an extension in the duration of cell proliferation in the caudal pole relative to the rostral pole. Accordingly, more neurons should be generated in the caudal pole by adulthood. The tangential expansion in the proliferative zone and a concomitant increase in progenitor cells in the caudal pole entail more radial columnar units superficial to its precursor pool (Rakic, 1974, 1995, 2002). These findings suggest that as overall neurogenetic schedules lengthen between species, the caudal cortex expands primarily in the number of radial columnar units. This argument is supported by the observation that neurons per unit of cortical surface area ether vary relatively little or increase in the caudal pole and that cortical areas in the caudal pole expand disproportionately relative to other cortical regions (Kaskan et al., 2005; Charvet et al., 2013).

In contrast, a shortened duration of neurogenesis entails that progenitor cells will grow fewer in numbers. A tangential expansion of the proliferative zone without a concomitant increase in proliferative cells entails a tangential expansion of the frontal cortex with fewer neurons generated within a radial columnar unit. This argument is supported by the observation that neurons per unit of cortical surface area and neuron densities in the rostral pole have a tendency to decrease as developmental schedules lengthen and isocortical neurons increase (Semendeferi et al., 2001; Sherwood et al., 2006; Collins et al., 2010; Spocter et al., 2012; Charvet et al., 2013; Finlay and Workman, 2013). For instance, the owl monkey (*Aotus trivirgatus*) contains approximately 70,000 neurons per unit of cortical surface area in the rostral region of the isocortex but a capuchin (*Cebus apella*) contains approximately 56,000 neurons in an equivalent region even though the capuchin isocortex has three times more neurons than the owl monkey (Charvet et al., 2013).

The developmental mechanisms accounting for the tangential expansion of the rostral cortical proliferative zone without a concomitant increase in progenitor cells are not clear. One possibility is that extending the duration of neurogenesis could force the cortical progenitor pool in the rostral region to expand tangentially because of growth of adjacent regions. That is, areas where cells undergo terminal neurogenesis late in development could grow substantially during development and force the growth of areas where cells undergo terminal neurogenesis earlier. The growth of regions adjacent to the rostral cortex (e.g., caudal cortex) might force the tangential expansion of the rostral cortical proliferative zone. Alternatively or in addition, post-proliferative cells superficial to the proliferative pool in the rostral cortex may force the tangential expansion of its proliferative zone. Species differences in the number and/or size of the post-proliferative cells shortly after neurogenesis onset could cause species differences in tangential expansion of the cortical proliferative zone. Mechanical effects acting on the kinetics of cell proliferation have been described previously (Smart, 1972; Shraiman, 2005; Striedter and Charvet, 2009). However, whether these mechanical explanations account for the tangential expansion in the rostral isocortex of species would need to be tested.

Differential growth patterns across the rostro-caudal axis of the proliferative zone could cause subsequent differences in growth trajectories via mechanical tension and by altering morphogenetic gradients across the cortical proliferative zone (Schwank and Basler, 2010; Janmey et al., 2013; Schluck et al., 2013). For instance, a reduced cell density in the rostral pole may be linked to a reduction in the expression of genes within the rostral cortical proliferative zone, which may have important downstream consequences on the graded expression of other genes across the isocortex. Altered gradients of expressed genes might influence cell cycle rates and/or levels of cell death (Aboitiz and Montiel, 2012; Aboitiz and Zamorano, 2013; Schluck et al., 2013), which may have an important influence on the gradients in isocortical developmental schedules across species.

### Conflict of interest statement

The author declares that the research was conducted in the absence of any commercial or financial relationships that could be construed as a potential conflict of interest.
